# Dental Reimplantation Treatment and Clinical Care for Patients with Previous Implant Failure—A Retrospective Study

**DOI:** 10.3390/ijerph192315939

**Published:** 2022-11-29

**Authors:** Jiakang Yang, Lixuen Siow, Xinyue Zhang, Yu Wang, Huiming Wang, Baixiang Wang

**Affiliations:** Stomatology Hospital, School of Stomatology, Zhejiang University School of Medicine, Zhejiang Provincial Clinical Research Center for Oral Diseases, Key Laboratory of Oral Biomedical Research of Zhejiang Province, Cancer Center of Zhejiang University, Hangzhou 310000, China

**Keywords:** dental implant failure, reimplantation, risk factors, patients’ satisfaction

## Abstract

(1) Objectives: This study evaluated the clinical outcomes of dental implants placed in previously failed sites and discussed the risk factors that mattered in reimplantation. (2) Methods: All the cases by one specific implantologist during his first five years of clinical practice were screened, with a focus on those who received reimplantation. The clinical outcomes were assessed, including the implant survival, peri-implant health, and patients’ satisfaction. (3) Results: 28 patients (31 implants) were recorded as failures from 847 patients (1269 implants), with a 2.4% overall failure rate at the implant level, of whom 19 patients (21 implants) received reimplantation treatment. After a mean follow-up of 33.7 ± 10.1 months (95% CI 29.1–38.3 months), 20 implants remained functional, but 1 implant revealed a secondary early failure, indicating a 95.2% overall survival rate. The mean probing depth (PD), modified sulcus bleeding index (mSBI), and marginal bone loss (MBL) of the surviving reinserted implants were 2.7 ± 0.6 mm (95% CI 2.5–3.0 mm), 0.7 ± 0.5 (95% CI 0.5–1.0), and 0.5 ± 0.6 mm (95% CI 0.3–0.8 mm), respectively. Embedded healing occurred more frequently in the reinserted implants than in the primary implants (*p* = 0.052). The patients’ satisfaction suffered from implant failure, but a successful reimplantation could reverse it with close doctor–patient communication. (4) Conclusions: Reimplantation treatment was recommended, based on a thorough evaluation and consideration of the risk factors combined with effective communication with the patients.

## 1. Introduction

We live in an ageing society. According to the fourth national oral survey in China, 4.5% of 65–74-year-old people present an edentulous jaw, with 22.5 teeth remaining, and 63.2% of the dentition defects have been restored with different restoration plans. With economic growth and an increased awareness of oral health issues, more and more elderly people with tooth loss are receiving dental implants in response to the 2030 Healthy China initiative.

Unfortunately, dental implant failures are also on the rise due to various reasons, including patient-related factors (systemic diseases, poor oral hygiene, etc.) and operator-related factors (poor clinical experience, aggressive surgical or prosthetic plan, etc.), which plague patients and implantologists. When an implant exhibits pathological loosening, spontaneous pain, pyorrhea, and irreversible surrounding-bone resorption, it must be removed [1]. Chranovic et al. discovered 642 cases of implant failure (6.36%) out of 10,096 implants, with 176 implants (1.74%) failing before a secondary surgery [2]. A one-year retrospective study also revealed a 4.2% (362/8540) early failure rate of implant restorations within one year of implantation [3].

It was recommended nearly 40 years ago to reinstall implants in sites where failed implants had been removed and healed well [4]. Grossman and Levin revealed the survival rate of 31 reimplanted implants and suggested that it was significantly lower than that of common implantation (70.97% vs. 93.08%), indicating a higher risk of reimplantation treatment [5]. According to Mardinger et al., the survival rate of 144 implant failures followed by reimplantation was 92.36%, with only one out of seven cases of the secondary reimplantation failing [6]. In Wang et al.’s study, a success rate of 90.6% and a cumulative survival rate of 94.6% were discovered in 67 cases of early reimplantation failure, with an average follow-up of 67 months [7].

In such situations, the implantologist’s clinical competence, experience, and personal habits (implant brand preference, subtle differences in surgery, etc.) can have an impact on the prognosis. It is challenging to rule out the interference of a single implantologist’s subjective factors in a retrospective study with a large sample of patients from multiple implantologists. In this study, the clinical cases from a certain implantologist during his first five years of clinical practice (1 January 2017 to 31 December 2021) were screened, and all the failed cases were thoroughly evaluated, including those who received the reimplantation. Based on the outcome of the implants placed in the previously failed sites, we preliminarily examined the possible influence of risk factors in reimplantation, including patient systemic and local factors, as well as flaws in treatment design and execution. In these complex and challenging clinical situations, which give the patients nonnegligible pain and trauma, additional attention was paid to the patients’ psychological status. We analyzed and evaluated the patients’ satisfaction with the co-located reimplantation and its changes throughout the treatment process. The purpose of this study was to evaluate the clinical outcome of dental implants placed in previously failed sites and discuss risk factors that matter in reimplantation. We hypothesized that reimplantation treatment could yield an acceptable clinical outcome based on a thorough evaluation, consideration of the risk factors, and effective communication with the patients. We hope that this study can inform the development of a young implantologist in dealing with consecutive emerging implant failures in the early career stage, which could provide a useful reference for their peers.

## 2. Materials and Methods

### 2.1. Inclusion and Exclusion Criteria

In this retrospective study, we searched all the cases of dental implant treatment delivered by one specific implantologist in the Affiliated Stomatology Hospital of Zhejiang University School of Medicine, from 1 January 2017 to 31 December 2021, and collected information from the patients who received reimplantation. The study was approved by the Ethics Committee of the Stomatology Hospital, Zhejiang University School of Medicine, China. The inclusion criteria were: (1) ≥18 years old, (2) patients who have had one or more implant failures and who have received reimplantation, and (3) patients in good general condition, with no major systemic diseases, classified into ASA (the American Society of Anesthesiologists) class I or II and are able to tolerate dental implant surgery (ASA Physical Status Classification System, 2020). The patients who met the following exclusion criteria were excluded: (1) those with uncontrolled systemic disease that impedes osseointegration, (2) those with untreated severe periodontitis of grade III or IV [8] or periodontal surgery in the past three months, (3) a history of head and neck radiotherapy within the last five years, (4) a history of bisphosphonate administration within the last five years, and (5) systemic use or oral local use of antibiotics in the past month.

### 2.2. Patients’ Information Collection

For the patients who met the inclusion or exclusion criteria, the following information was collected: (1) gender, age (at the time of primary implantation), (2) general health status [8], history of systemic diseases, history of medication use. (3) history of confirmed and treated periodontal disease [9], (4) use of tobacco, bruxism [10], and unilateral masticatory habit, (5) oral hygiene habits (tooth brushing methods and flossing), (6) timing of key treatment points (primary implantation, removal of failed implant, reimplantation, and final restoration), (7) condition of soft and hard tissues at implant sites, (8) details of the implants for the primary implantation and reimplantation (brand, length, diameter, etc.), (9) procedures for primary implantation and reimplantation, (10) and reasons why certain patients refused reimplantation treatment.

### 2.3. Reimplantation Treatment

#### 2.3.1. Removal of Failed Implants

During the post-primary implantation follow-up, the implants that met the above-mentioned failure criteria were removed, followed by site debridement. For the failed implants that fell out or could be easily removed by a needle holder, the residual inflammatory granulation tissue was scraped under anesthesia and the site thoroughly rinsed with 1% iodophor solution, and cefuroxime tablets (0.25 g × 2 per day) and tinidazole capsules (0.5 g × 2 per day) were administered orally for at least 3 days. For those that lost partial osseointegration but remained in situ, or that fractured with parts left in the bone, the failed implants were removed surgically. After a routine pre-surgical disinfection and preparation, a full mucoperiosteal flap was elevated under local anesthesia and the surrounding bone was cut with a high-speed trephine drill, if necessary, until the implant could be screwed or clamped out. Osteotomy (expanded removal of infected bone) was performed when obvious infection was detected. Then, the site was thoroughly rinsed with 1% iodophor stock solution + 3% hydrogen peroxide in turns. A guided bone regeneration (GBR) with Bio-Oss bone grafts and Bio-Guide absorbable collagen membranes was performed to promote the bone regeneration in the large bone defects (>5 mm). Cefuroxime (1.5 g per day) and tinidazole (1.6 g per day) were administered intravenously after surgery for at least 3 days to prevent infection.

#### 2.3.2. Reimplantation Surgery

After 3–6 months of healing, the patients were carefully reviewed and assessed by Cone Beam Computed Tomography (CBCT) (NewTom3G, QR, Verona, Italy) before the reimplantation treatment. On the basis of an evaluation of the possible reasons for the primary failures, the implantologist might make necessary adjustments to the treatment plan with the patients’ informed consent, with adequate communication, in order to move on to the next stage of treatment in a positive doctor–patient relationship. The adjustments include but are not limited to: (1) fine-tuning of the reimplantation site, (2) change of implant type, length or width, (3) use of implants with better mechanical properties, more complex surface treatment, or greater self-tapping ability, (4) change in the way the implant heals (embedded or emerged), (5) use of different bone augmentation materials, (6) change in the restoration plan. The sutures were removed 7 days after the implantation, and the patients were reviewed 3–6 months later with regular CBCT scans. A second-stage exposure was performed on the well-osseointegrated implants, which would be performed at the same time or 2 weeks later. For the implants in the aesthetic zone, provisional prostheses were delivered for 1–6 months, until the soft tissue reached a satisfying stable contour, before the final restoration. For the patients with All-On-4 or All-On-6 full-arch dental rehabilitation, immediate or early prostheses were delivered if the implants had sufficient primary stability [11] and would be re-inserted by final restoration after 6 months.

### 2.4. Follow-Up

#### 2.4.1. Clinical and Radiographic Examination

A routine follow-up was set at 1 month, 3 months, 6 months, and 12 months post-restoration, with additional re-examinations when the patients reported any discomfort. The clinical examination included: implant motility, percussion response, mucosal redness, swelling, tenderness, and peri-implant pathological exudate. According to the 2007 Pisa Consensus Conference of the International Congress of Oral Implantologists (ICOI), the prognosis of dental implants is divided into four categories: success, satisfactory survival, compromised survival, and failure [1]: (1) Success: no pain or tenderness on function, no mobility, less than 2 mm of marginal bone loss (MBL) compared to after the primary implantation, no exudate. (2) Satisfactory survival: no pain or no motility on function, 2–4 mm MBL, no exudate. (3) Compromised survival: possible sensitivity on function, no mobility, MBL greater than 4 mm but less than 1/2 the length of the implant, probing depth (PD) greater than 7 mm, and occasional exudate. (4) Failure (all of the following): pain on function, mobility, MBL greater than 1/2 the length of the implant, uncontrolled exudate, dislocation. Six peri-implant sites (distal-buccal, buccal, mesial-buccal, distal-lingual, lingual, mesial-lingual) were probed and the PD was recorded, together with the modified Sulcus Bleeding Index (mSBI) [12]: 0 = no bleeding when the periodontal probe is passed along the peri-implant gingival margin; 1 = isolated bleeding spots; 2 = blood forms a confluent red line on the margin; 3 = profuse or spontaneous bleeding. MBL was defined as the linear distance (in mm) measured at both the proximal and distal mesial planes from the most coronal point of the surrounding bone to the cervical plane of the implant. The objective measurements were performed by two independent experienced dentists and the mean value of the results was recorded.

#### 2.4.2. Patients’ Satisfaction Assessment

The Visual Analogue Scale (VAS) [13] was utilized to assess the patients’ satisfaction at key points along the whole treatment process, and to analyze the psychological changes of the patients, who were instructed to recall their overall satisfaction with their treatment at the final follow-up (Appendix A).

### 2.5. Statistical Analysis

Graphpad Prism 9.0 was used for analyzing the data. The measures were described in the form of the mean ± standard deviation and a 95% confidence interval (95% CI). The overall survival rate and success rate of the reimplanted implants were calculated. A paired Student’s t-test was used to compare the differences in the continuous variables between the two groups, while an ANOVA test was performed with more than two groups (Tukey’s for further multiple comparisons). Fisher’s exact test or the chi-squared test was used to evaluate the categorical variables. *p* < 0.05 was considered statistically significant.

## 3. Results

### 3.1. Patients’ General Information

**Figure 1:**
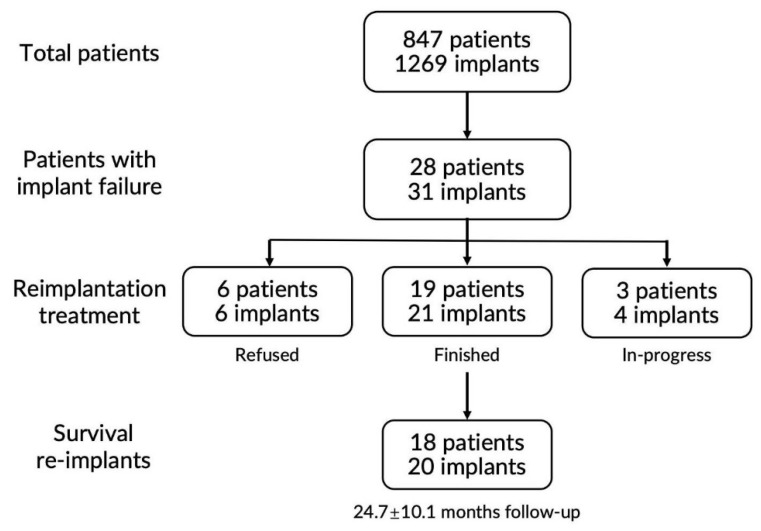
The distribution of patients with implant failure.

From 1 January 2017 to 31 December 2021, the attending implantologist treated 847 patients with partial or total edentulism and implanted 1269 implants, of which 31 implants (28 patients) were recorded as implant failures, with a 2.4% overall failure rate at the implant level. Implant failure was identified at a mean of 10.2 ± 11.0 months (0–33.5 months, 95% CI 6.1–14.3 months) after the primary implantation, with 20 (64.5%) implants defined as early failures and 11 (35.5%) implants as late failures. Up to the end of 2021, 19 patients received reimplantation, totaling 21 implants, with one patient experiencing three implant failures and reimplantations (Appendix B). At this time, three patients had not completed the reimplantation treatment. Otherwise, six patients refused reimplantation attempts after the primary failure with various considerations (Appendix C), while one patient refused the second reimplantation after implant failure was detected again after the first reimplantation (#10 patient). The distribution of patients with implant failure was summarized in Figure 1.

Of the 19 patients who underwent reimplantation (11 men, 8 women), the mean age was 45.7 ± 16.3 years (21–80 years, 95% CI 37.9–53.6 years) at the primary implantation. The mean interval between the removal of the 21 failed implants and the reimplantation was 6.7 ± 3.4 months (0–15 months, 95% CI 5.0–8.3 months), including 1 implant (one patient), which was reinserted immediately. One patient (one implant) withdrew from treatment because of a second early failure, and the other 18 patients (20 implants) received rehabilitation after a mean 6.4 ± 3.9 months (0.5–18 months, 95% CI 4.5–9.4 months) of healing.

### 3.2. General Outcome of Reimplantation Treatment

With a mean follow-up of 33.7 ± 10.1 months (95% CI 29.1–38.3 months) after the reimplantation surgery, 20 implants remained functional while 1 implant failed before the second-stage exposure. Within the 20 surviving implants, the mean peri-implant PD was 2.7 ± 0.6 mm (95% CI 2.5–3.0 mm), with 13.3% of loci showing a PD greater than 4 mm, while the mSBI was 0.7 ± 0.5 (95% CI 0.5–1.0), with 0.1% of loci showing an mSBI greater than 3 mm. The mean MBL (both mesial and distal) on the radiographic images was 0.5 ± 0.6 mm (95% CI 0.3–0.8 mm), with the MBL of one implant being greater than 2 mm (but less than 4 mm). Therefore, 1 implant failed and 1 implant was detected as a satisfactory survival, while the other 19 implants showed as a success, resulting in an overall survival of the reinserted implants of 95.2% and an overall success rate of 90.4%, with a mean follow-up of 33.7 ± 10.1 months.

### 3.3. Systemic and Local Conditions of Reimplanted Patients

Among the 19 reimplanted patients, 3 (15.8%) had mild hypertension (140–159 mmHg systolic pressure or 90–99 mmHg diastolic pressure) at the first visit; 10 (52.6%) had been diagnosed with stage III-IV chronic periodontitis at the implant site, in the adjacent teeth, or throughout the mouth; and only 5 (26.3%) reported a routine periodontal treatment history. Three (15.8%) patients self-reported a smoking history for more than 10 years (at least 10 cigarettes per day) prior to the primary implantation, while 13 (68.4%) had a unilateral masticatory habit for more than 3 years. After the primary implantation, 8 (42.1%) learned the Bass brushing technique recommended by the American Dental Association, 12 (63.2%) flossed regularly, and 7 (36.8%) adapted additional oral hygiene measures, including gargling. Four of the nineteen reimplanted patients were considered to have probable sleep bruxism, one of whom had a protective polyethylene terephthalate occlusal guard perforated on their provisional prothesis after reimplantation, indicating a definite bruxism.

Of a total of 21 reimplanted sites, 3 (14.3%) had Class I bone; 7 (36.8%) had Class II bone; 8 (42.1%) had Class III bone; and 3 (14.3%) had Class IV bone according to the canonical Lekholm and Zarb classification [14]. Only one alveolar site was detected with a pathological infection and bone resorption on the CBCT images, but it still received an immediate reimplantation after a thorough debridement (patient #17). Anatomically, the 21 replaced implants were distributed in the maxillary anterior area (1, 4.8%), maxillary posterior area (7, 36.8%), mandibular anterior area (4, 21.1%), and mandibular posterior area (9, 47.4%).

### 3.4. Reimplantation Surgery and Rehabilitation

All of the implants used for the primary implantation and reimplantation were threaded titanium implants in cylindrical or tapered columnar shapes with sandblasted, acid-etched surfaces, which were provided by certain manufacturers including ITI (Straumann AG, Waldenburg, Switzerland), Nobel (Nobel Biocare, Yorba Linda, CA, USA), and ZDI (Zhejiang Guangci Medical Devices, Ningbo, China). The average length and diameter of the primary implants were 11.2 ± 1.8 mm (8.0–16.0 mm, 95% CI 10.4–12.1 mm) and 4.1 ± 0.5 mm (3.3–5.0 mm, 95% CI 3.8–4.3 mm), while those of the reinserted implants were 11.1 ± 1.3 mm (10.0–13.0 mm, 95% CI 10.5–11.7 mm) and 4.0 ± 0.5 mm (3.3–4.8 mm, 95% CI 3.7–4.2 mm), with no statistically significant difference (Table 1).

Of the 21 primary implants, 11 (52.4%) underwent emerged healing and the other 10 (47.6%) underwent embedded healing. In the reimplantation treatment, there were 4 (56.0%) with emerged healing and the other 17 (44.0%) with embedded healing, which did not show a significant difference (Table 1). In the primary implantation, four (21.1%) patients underwent a trans-alveolar sinus floor elevation; one (5.3%) patient underwent a lateral-window sinus floor elevation; and bone condensation was performed in one (5.3%) patient. Similarly, in the reimplantations, there were three (15.8%) patients with a trans-alveolar sinus floor elevation and two (10.5%) with a lateral-window sinus floor elevation, and another two (10.5%) with a GBR. In terms of prostheses, a single crown was the main type in 14 (73.7%) patients, and short bridges were delivered to 2 (10.5%) patients, while 3 (15.8%) patients underwent whole-arch fixed restoration (“All-On-4” or “All-On-5”).

### 3.5. Patients’ Satisfaction

**Figure 2:**
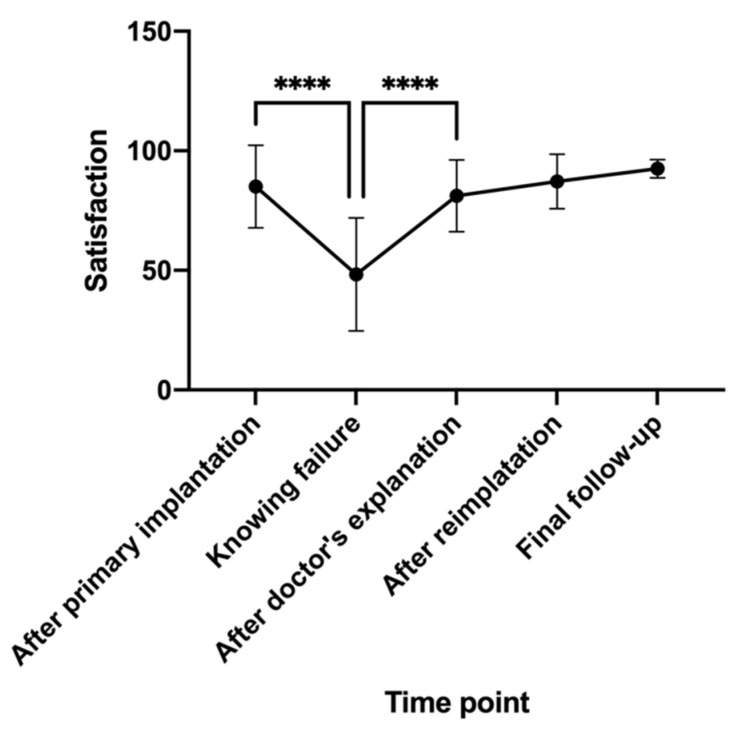
Reimplanted patients’ satisfaction. **** *p* < 0.0001, by Tukey’s multiple comparisons.

This study examined the satisfaction of 18 reimplanted patients with successful restoration, functioning for more than 6 months at the last follow-up, excluding the #10 patient with a secondary failure. The mean satisfaction score of the patients was 85.1 ± 17.3 (95% CI 76.5–93.7) after the primary implantation but fell dramatically to 48.3 ± 23.6 (95% CI 36.5–60.0) upon knowing that the implants had failed (*p* < 0.0001). However, it rose back to 81.2 ± 15.0 (95% CI 73.7–88.6) after communication and explanation by the implantologist, which was significantly higher (*p* < 0.0001). The mean satisfaction score remained at 87.2 ± 11.4 (95% CI 81.5–92.8) after the reimplantation surgery and 92.5 ± 3.8 (95% CI 90.6–94.4) at the last follow-up (Figure 2).

## 4. Discussion

The causes of implant failures are usually a combination of factors, with a relatively low incidence (2.4% in this study) and serious consequences when they do occur. The reasons for early implant failure include medical factors (mainly related to the operators), such as an immediate/early loading with inadequate primary stability, clinician inexperience, and osteonecrosis due to intraoperative heat production, and patient-related factors such as systemic diseases, poor oral hygiene, and severe periodontitis. While a late implant failure can be attributed to an occlusal overload, adhesive residue, poor restoration design, etc. (operator-related factors), excessive occlusal forces, poor oral hygiene, an inflammation of adjacent teeth, etc. (patient-related factors) can also be responsible [15,16]. There are a number of behavioral patterns patients exhibit when implants fail, including but not limited to: (1) opting for a reimplantation attempt, (2) choosing other restoration means, (3) opting for other treatment modalities (fixed partial denture, removable denture, etc.), (4) abandoning reimplantation due to high risks or objective circumstances that do not meet the requirements for reimplantation, (5) losing confidence and terminating the treatment. Regardless of the patient’s behavior, the goal of improving the prognosis for the patient’s subsequent treatment and increasing patient satisfaction should always be at the heart of the implantologist’s decision.

In this study, 28 patients experienced failure after the primary implantation. The reimplantation was abandoned by six (21.4%) patients, indicating that reimplantation was relatively well-received by the majority of patients. The main reasons the patients chose to forgo reimplantation varied (Appendix C). Here, 19 reimplanted patients with a total of 21 implants revealed an overall survival rate of 95.2% and an overall success rate of 90.4%, with a mean follow-up of 24.7 ± 9.8 months, which was relatively acceptable based on the large-scale systematic reviews [17,18]. No unanimous opinion has been reached regarding the prognosis of reimplantation. Gomes et al. systematically reviewed and meta-analyzed the clinical literature related to reimplantation and showed that the implant reimplantation survival rates (88.7%) and survival rates for second-attempt reimplantations (67.1%) were lower than those for conventional non-reimplantable implants [19]. On the other hand, a study by Wang et al. showed a high success rate (90.6%) and cumulative survival rate (94.6%) in 67 reinserted implants at an average of 67 months after surgery, which might be associated with the early failures of primary implants [7]. In this study, a total of 20 reinserted implants completed the restoration and kept on functioning for 16.5 ± 8.7 months postoperatively, showing no pain, tenderness, or pathological exudate. The mean peri-implant PD was 2.7 ± 1.0 mm; the mean mSBI was 0.7 ± 0.7; and the mean MBL was 0.5 ± 0.6 mm, with only one implant showing MBL greater than 2 mm but less than 4 mm (in #6). All the results indicated an overall healthy state of the peri-implant soft and hard tissues of the re-inserted implants. According to Wang et al., 67 early failed sites were reimplanted and exhibited a mean 1.7 ± 1.3 mm MBL, with 1 implant’s MBL being 4 mm and 3 implants’ MBL being 2–4 mm at a mean 69.4 month follow-up [5]. Nevertheless, significant changes in MBL were observed between 12 months and 24 months postoperatively, and at 24 months compared to 36 months postoperatively, suggesting some active remodeling of the peri-implant bone tissue after reimplantation [20]. Besides the above-mentioned results, in further studies, more objective evaluation criteria are recommended to assess the prosthodontic outcome of reimplantation comprehensively, such as the functional implant prosthodontic score (FIPS) [21].

According to this study, there was no statistical association between the prognosis of reimplantation and a number of conventional risk factors, including age, gender, smoking habits, history of periodontitis, edentulism, and oral hygiene. In clinical practice, most implant failures cannot be attributed to a single factor or even if the cause was ambiguous, which would emerge in the reimplantation phase. In terms of systemic diseases, only three patients reported mild hypertension in our study. As a growing number of old patients with systemic diseases, including type 1 diabetes [22], receive dental implant treatment, the influence of systemic diseases on the prognosis of dental reimplantation needs further large-sample clinical studies. Interestingly, bruxism was a factor that attracted attention in the reimplantation treatment. Bruxism is a relative explicit risk factor in dental implant treatment and can be managed by prosthodontics [23]. Here, we recommend a protective polyethylene terephthalate occlusal guard as an appropriate and cost-effective intervention.

**Figure 3:**
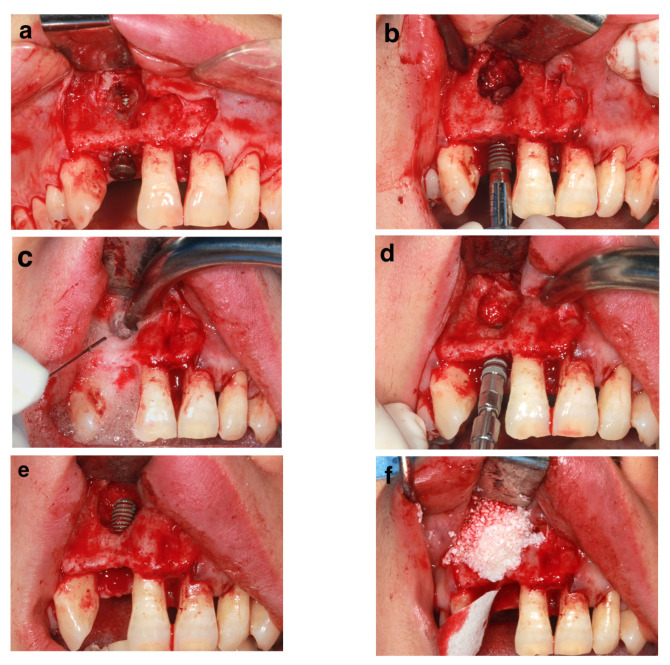
An Immediate reimplantation surgery (patient#17). (**a**) Primary implant failed with buccal bone fenestration. (**b**) Removal of failed implant. (**c**) Debridement of implant cavity with 3% H_2_O_2_ rinse. (**d**) and (**e**) Replacement with a new implant immediately. (**f**) Guided bone regeneration with heterologous bone grafts and an absorbable collagen membrane.

Although dental implant failure prolonged the time of edentulism and loss of occlusal support, which could deteriorate the health of patient’s masticatory system [24], we still recommend a conventional or late timing for the reimplantation, allowing the hard and soft tissues to heal uneventfully (at least 12 weeks post-removal of the failed implant [25], and removing as many of the detrimental physiological influences from the primary failure as possible. However, in a few cases, such as in the aesthetic zone, immediate or early reimplantation could be chosen to shorten the treatment period, to better preserve the bone volume, and reduce the atrophy of the alveolar bone caused by edentulism [26]. The average interval between the removal of the failed implant and reimplantation was 6.7 ± 3.3 months, with only one patient receiving immediate reimplantation (patient #17). With relatively good conditions (>2 mm intact cervical buccal bone and thorough debridement), the immediately re-inserted implant yielded a successful clinical outcome 46 months later (Figure 3).

No statistical differences were found when comparing the lengths and diameters of the reinserted implants with the primary implants in this study, and most of the reinserted implants were the same length and diameter as the primary implants. The length and diameter of the implant had an important impact on its osseointegration and initial osseointegration area [27], as well as its mechanical strength [28]. In the study by He et al., all 15 reinserted implants were significantly larger in diameter than the primary implants, which suggested that wider implants might be beneficial to the prognosis of reimplantation [29]. Choosing a wider implant to be reinserted was also recommended by some authors [5,30]. However, the decision on the implant’s physical parameters should be based on the local site situation itself, the volume of available bone, and the anticipated biting force of the patient. A longer or wider implant could be chosen in some cases to provide better primary stability, such as in immediate reimplantation. When the primary implant failed because of a fracture, a wider implant was recommended to resist possible abnormal loading, especially for some middle-aged men with robust biting muscles.

Despite no statistical difference, in this study, the re-inserted implants were more in embedded healing than the primary implants, which implies the implantologist’s conservative decision-making in terms of the implant healing. Generally, when the primary stability of an implant is relatively adequate (torque ≥ 35 Ncm), a healing abutment can be delivered to accelerate the shaping of the soft tissues. Healing abutments can accelerate the shaping of soft tissues, but when eating and chewing, it is inevitable that the healing abutment will be compressed and the load will be transferred rigidly to the implant–bone interface. Animal studies have found that a 10 N static immediate load can increase the proportion of poorly structured new bone during the initial healing phase of a dental implant, which is detrimental to the functional loading of the implant [31]. We observed that placing implants in the mandibular anterior region puts them at a higher risk of pathological micromotion resulting from tongue movement. Two patients (#7 and #18) definitely reported that they could not help licking the healing abutments after the primary implantation. A retrospective study by Zhang et al. on early failure found that simultaneous application of healing abutments and bone grafting resulted in a significantly higher risk of early failure [32]. We recommended embedded healing as a way to provide an uneventful osteointegration for re-inserted implants.

Without a complex questionnaire design, the VAS method allowed patients to recall their overall satisfaction at key time points from the primary implantation without deep consideration, and they could write on the VAS scales to present the level of satisfaction with the least amount of recall bias. Interestingly, the mean satisfaction of the 18 reimplanted patients dropped from 85.1 ± 17.3 post-primary implantation to 48.3 ± 23.6 when they knew the implant failure had occurred, but the mean score soared back to 81.2 ± 15.0 after the implantologist’s meticulous explanation and comfort, with both changes showing statistical significance. Then, the satisfaction level remained over 80 with slight increases after the reimplantation and successful restoration. This dramatic development indicated that implant failure did largely influence the patient’s satisfaction with the implant treatment, but it can be reversed by the implantologist’s explanation, and most essentially, successful reimplantation. Since the failure of surgical procedures can have a significant psychological impact on patients [33], when re-treating, medical decisions and communication strategies must be more sensitive to the patient’s psychological state. Therefore, it is crucial to actively reassure the patient and create a psychological development process. Therefore, we recommend some tips for implantologists when implant failure occurs: (1) Acknowledge that implant failure does happen and try to accept all the patients’ complaints; at first, be helpful in calming the patient’s possible agitation and anger. (2) Meticulously explain all the possible reasons impartially, but do NOT purely attribute them to either the patient or the implantologist. Blaming the patient can exacerbate the conflict, while blaming the implantologist can undermine the patient’s trust in the implantologist. (3) Cooperate with the patient in the further treatment planning and respect the patient’s final decision (no matter what the outcome). A collaborative model of patient–clinician communication promotes better clinical outcomes.

This study included all the reimplantation treatment cases in the first 5 years of the implantologist’s career in implant dentistry, which was informative to some extent. The majority of patients came from Hangzhou or other cities and counties in the province with relatively high economic development and a favorable attitude toward dental implant treatment. Prior to taking up autonomous clinical practice, the implantologist, with a PhD degree, had finished a 3-year nationwide standardized pre-clinical training course and a 1-year specialized internship, directly supervised by a senior implantologist. This provided him with a relatively solid theoretical foundation and basic clinical experience, which is currently the mainstay of doctor training in China’s AAA stomatology hospital. The clinical outcomes of all the patients who had received reimplantation by such a single implantologist could provide some guidance for young implantologists in dealing with implant failure cases.

The popularity of dental implant treatment stems from the high economic level and the unavoidable commercialization of the promotion. However, the relatively high cost of implant restorative treatment invariably affects patients’ expectations of implant treatment outcomes. The failure of implantation can seriously undermine a patient’s trust in the treatment and the implantologist, which will put enormous pressure on implantologists to try a second implantation attempt, making the cases more complex. The question of how to properly deal with dental implant failure and improve the prognosis of reimplantation treatment still requires more investigation with large-scale samples and longer follow-up.

## 5. Conclusions

Based on a thorough evaluation and consideration of the risk factors, reimplantation treatment is recommended in combination with effective communication with the patients. We suggest taking a conservative embedded healing approach in the reimplantation, and additional clinical care should be given to patients, especially in the psychological aspects. Successful reimplantation could be a direct and sufficient treatment modality for dental implant failure, which would serve to promote the global population’s oral health.

## Figures and Tables

**Table 1:** Characteristics of length, diameter, and healing of primary implants and reinserted implants.

	Primary Implantation (95% CI)	Reimplantation (95% CI)
Implant parameters		
Length (mm) ^a^	11.2 ± 1.8 (10.4–12.1)	11.1 ± 1.3 (10.5–11.7)
Diameter (mm) ^b^	4.1 ± 0.5 (3.8–4.3)	4.0 ± 0.5 (3.7–4.2)
Healing way ^c^		
Emerged	11	4
Embedded	10	17

^a^: *p* = 0.619 by paired t-test. ^b^: *p* = 0.356 by paired t-test. ^c^: *p* = 0.052 by Fisher’s exact test.

**Figure A1:**
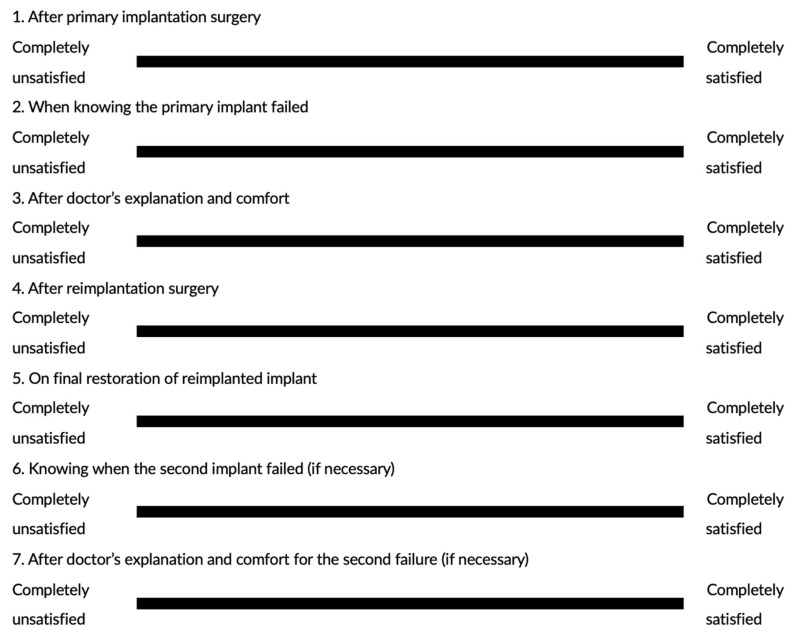
Visual Analogue Scale (VAS) of patient’s satisfaction. On the scales of the VAS form, the left end represented complete dissatisfaction while the right end represented complete satisfaction. Based on their primary impressions, patients could inscribe a point on each scale. They are assured that any unsatisfactory results will have no impact on subsequent treatment. The exact scores of satisfactions were measured as the length of the inscribed point to the left end (if it was at half, it represented a satisfaction level of 50/100).

## Data Availability

Not applicable.

## Appendix B

**Table A1:** Summary of information of the 19 reimplanted patients.

Number	Gender	Age (Year)	Systemic Condition	Replanted Sites	Failure Time after Primary Implantation (Months)	Failure Type	Failure Reason of Primary Implant	Stage III/IV Periodontitis
1	Female	73	Well	26	4.5	Early	Poor osteointegration	III
2	Male	46	Well	36	24.5	Late	Abutment screw fracture	0
3	Male	49	Well	34	4.0	Early	Poor osteointegration	0
4	Male	40	Well	35	3.5	Early	Infection	0
5	Male	35	Well	16	6.0	Early	Poor osteointegration	0
6	Male	41	Well	32, 35, 42 (All-On-4)	27. 5 (all)	Late	Implant fracture (all)	IV
7	Female	80	Well	32	1.0	Early	Poor osteointegration	IV
8	Male	64	Mild hypertension	36	3.0	Early	Poor osteointegration	0
9	Female	21	Well	46	2.0	Early	Infection	0
10	Female	26	Well	15	4.0	Early	Poor osteointegration	0
11	Male	60	Mild hypertension	14 (All-On-4)	12.0	Late	Implant malposition	IV
12	Male	33	Well	43	1.5	Early	Poor osteointegration	0
13	Male	24	Well	15	5.0	Early	Poor osteointegration	0
14	Female	33	Well	34	4.5	Early	Deficient alveolar bone	0
15	Female	26	Well	47	15.0	Late	Peri-implantitis	0
16	Female	54	Well	16	7.0	Early	Poor osteointegration	III
17	Female	43	Well	12	1.0	Early	Implant malposition	III
18	Male	49	Mild hypertension	44	3.0	Early	Poor osteointegration	IV
19	Male	62	Well	14 (All-On-4)	2.0	Early	Bruxism	IV

**Table A2:** Summary of information of the 19 reimplanted patients.

Number	Primary Implant (mm)	Healing Way of Primary Implant	Bone Augmentation of Primary Implantation	Time Interval between Reimplantation and Removal of Failed Implant
1	Nobel PMC 4.3 × 10	Embedded	Trans-alveolar sinus floor elevation	4.5
2	ZDI BL 4.8 × 10	Embedded	None	5.0
3	ZDI BL 3.3 × 12	Embedded	Bone condensation	7.0
4	Nobel PMC 4.3 × 10	Emerged	None	11.5
5	ITI BL 4.8 × 10	Embedded	Trans-alveolar sinus floor elevation	10.5
6	Nobel Active 3.5 × 13 (32, 42), 4.3 × 13 (35)	Emerged (all)	None	10.5 (all)
7	Nobel PMC 3.5 × 11.5	Emerged	None	6.5
8	Nobel PMC 4.3 × 11.5	Embedded	None	15.0
9	ITI SLActive BL 4.1 × 10	Embedded	None	4.0
10	Nobel PMC 3.5 × 10	Embedded	Trans-alveolar sinus floor elevation	7.0
11	Nobel Active 5.0 × 13	Emerged	None	5.5
12	Nobel CC 3.5 × 16	Embedded	None	6.5
13	Nobel PMC 3.5 × 10	Embedded	Trans-alveolar sinus floor elevation	6.5
14	Nobel PMC 3.5 × 10	Embedded	None	7.0
15	Nobel PMC 4.3 × 10	Emerged	None	8.0
16	ZDI SP 4.8 × 8	Emerged	Lateral-window sinus floor elevation	4.5
17	ZDI BL 4.0 × 12	Emerged	None	0.0
18	ITI BL SLActive 4.1 × 10	Emerged	None	4.0
19	Nobel PMC 4.3 × 10	Emerged	None	3.0

**Table A3:** Summary of information of the 19 reimplanted patients.

Number	Replanted Implant (mm)	Healing Way of Replanted Implant	Bone Augmentation of Primary Reimplantation	Restoration Time after Reimplantation	Prosthesis
1	Nobel PMC 4.3 × 10	Embedded	Lateral-window sinus floor elevation	10.5	Single crown
2	ITI BL 4.8 × 10	Embedded	None	5.5	Single crown
3	ITI BL 3.3 × 12	Embedded	None	5.5	Single crown
4	Nobel PMC 4.3 × 10	Embedded	None	5.5	Single crown
5	ITI BL 4.8 × 10	Embedded	Trans-alveolar sinus floor elevation	6.5	Single crown
6	Nobel PMC 3.5 × 13 (32, 42), 4.3 × 13 (35)	Emerged (all)	None	0.5 (all)	All-On-5
7	Nobel PMC 3.5 × 11.5	Embedded	None	5.0	Bridge
8	ITI BL 4.1 × 10	Embedded	None	6.0	Single crown
9	ITI TiZr 3.3 × 10	Embedded	None	2.5	Single crown
10	Nobel PMC 4.3 × 10	Embedded	Trans-alveolar sinus floor elevation	Failed again	Single crown
11	Nobel Active 3.5 × 13	Emerged	None	2.0	All-On-4
12	Nobel CC 3.5 × 10	Embedded	Guided bone regeneration	18.0	Bridge
13	ITI BL 4.1 × 10	Embedded	Trans-alveolar sinus floor elevation	7.0	Single crown
14	ITI BL 3.3 × 12	Embedded	None	9.0	Single crown
15	Nobel PMC 4.3 × 10	Embedded	None	4.5	Single crown
16	ITI SP 4.1 × 10	Embedded	Lateral-window sinus floor elevation	8.0	Single crown
17	ZDI BL 4.0 × 12	Embedded	Guided bone regeneration	8.0	Single crown
18	ITI BL SLActive 4.1 × 10	Embedded	None	8.5	Single crown
19	Nobel PMC 4.3 × 10	Embedded	None	3.0	All-On-4

**Table A4:** Summary of information of the 19 reimplanted patients.

Number	Follow-Up Time (Months)	Mean Probing Depth (mm)	Mean Modified Sulcus Bleeding Index (mm)	Mean Marginal Bone Loss (mm)	Bruxsim	Smoking (>10 Cigarettes per Day)
1	33	3.0	1.2	0.8	Denied	None
2	26	2.0	0.0	0.2	Probable	None
3	26	3.6	1.0	0.2	Denied	None
4	26	3.0	2.0	0.6	Denied	None
5	17	2.2	0.7	0.2	Denied	None
6	19 (all)	2.3	0.8	1.2	Probable	None
7	31.5	3.0	1.0	0.2	Denied	None
8	11.5	1.8	0.3	0.2	Denied	>40 years
9	45.5	2.5	1.0	0.1	Denied	None
10	4 (failed again)	N.A.	N.A.	N.A.	Denied	None
11	29	3.2	1.2	0.6	Denied	10 years
12	31.5	2.3	0.5	0.2	Denied	None
13	24.5	2.7	0.2	0.6	Denied	None
14	24	2.5	0.2	0.4	Denied	None
15	19	1.8	0.7	0.3	Denied	None
16	29.5	3.0	0.0	1.1	Denied	None
17	46	3.2	0.3	0.8	Denied	None
18	26	4.1	1.3	0.5	Denied	None
19	12.5	2.8	1.0	0.3	Definite	>30 years

## Appendix C

**Table A5:** Summary of information of patients who out of reimplantation.

Number	Gender	Age (Year)	Systemic Condition	Failed Sites	Failure Time after Primary Implantation (Months)	Failure Type	Failure Reason of Primary Implant	Stage III/IV Periodontitis
1	Male	47	Well	31	4.0	Early	Poor osteointegration	III
2	Male	46	Well	41	5.0	Early	Poor osteointegration	III
3	Female	53	Well	36	Immediately	Early	Implant malposition (violating inferior alveolar nerve)	0
4	Female	32	Well	11	1.5	Early	Poor osteointegration	0
5	Male	53	Well	31	1.5	Early	Poor osteointegration	III
6	Female	37	Well	35	27	Late	Peri-implantitis	0

**Table A6:** Summary of information of patients who out of reimplantation.

Number	Primary Implant (mm)	Healing Way of Primary Implant	Bone Augmentation of Primary Implantation	Reasons Why Out of Reimplantation	Alternative Solution of Edentulism
1	Nobel PMC 3.5 × 11.5	Embedded	Bone condensation	Economically sensitive	Maryland fixed bridge
2	ITI BL 3.3 × 12	Embedded	GBR	Appropriate alternative restoration was recommended	Cantilever fixed bridge on the next implant (31)
3	ITI TiZr 3.3 × 10	Embedded	None	Severe complication destroyed patient’s confidence	None
4	Nobel CC 3.5 × 13	Emerged	GBR	Poor alveolar bone condition	Cantilever fixed bridge on the next implant (21)
5	ITI BL 3.3 × 12	Embedded	None	Economically sensitive	None
6	Nobel CC 3.5 × 8	Emerged	None	Inconvenience in treatment due to CoVID-19	None

## References

[1] Misch C.E., Perel M.L., Wang H.L., Sammartino G., Galindo-Moreno P., Trisi P., Steigmann M., Rebaudi A., Palti A., Pikos M.A. (2008). Implant success, survival, and failure: The International Congress of Oral Implantologists (ICOI) Pisa Consensus Conference. Implant Dent..

[2] Chrcanovic B.R., Kisch J., Albrektsson T., Wennerberg A. (2016). Factors Influencing Early Dental Implant Failures. J. Dent. Res..

[3] Carr A.B., Arwani N., Lohse C.M., Gonzalez R.L.V., Muller O.M., Salinas T.J. (2019). Early Implant Failure Associated with Patient Factors, Surgical Manipulations, and Systemic Conditions. J. Prosthodont..

[4] Adell R., Lekholm U., Rockler B., Brånemark P.I. (1981). A 15-year study of osseointegrated implants in the treatment of the edentulous jaw. Int. J. Oral Surg..

[5] Grossmann Y., Levin L. (2007). Success and survival of single dental implants placed in sites of previously failed implants. J. Periodontol..

[6] Mardinger O., Ben Zvi Y., Chaushu G., Nissan J., Manor Y. (2012). A retrospective analysis of replacing dental implants in previously failed sites. Oral Surg. Oral Med. Oral Pathol. Oral Radiol..

[7] Wang F., Zhang Z., Monje A., Huang W., Wu Y., Wang G. (2015). Intermediate long-term clinical performance of dental implants placed in sites with a previous early implant failure: A retrospective analysis. Clin. Oral Implants Res..

[8] James P.A., Oparil S., Carter B.L., Cushman W.C., Dennison-Himmelfarb C., Handler J., Lackland D.T., LeFevre M.L., MacKenzie T.D., Ogedegbe O. (2014). 2014 evidence-based guideline for the management of high blood pressure in adults: Report from the panel members appointed to the Eighth Joint National Committee (JNC 8). JAMA.

[9] Tonetti M.S., Greenwell H., Kornman K.S. (2018). Staging and grading of periodontitis: Framework and proposal of a new classification and case definition. J. Periodontol..

[10] Lobbezoo F., Ahlberg J., Glaros A.G., Kato T., Koyano K., Lavigne G.J., de Leeuw R., Manfredini D., Svensson P., Winocur E. (2013). Bruxism defined and graded: An international consensus. J. Oral Rehabil..

[11] Penarrocha-Diago M., Penarrocha-Diago M., Zaragozí-Alonso R., Soto-Penaloza D., On Behalf of the Ticare Consensus Meeting (2017). Consensus statements and clinical recommendations on treatment indications, surgical procedures, prosthetic protocols and complications following All-On-4 standard treatment. 9th Mozo-Grau Ticare Conference in Quintanilla, Spain. J. Clin. Exp. Dent..

[12] Mombelli A., Lang N.P. (1994). Clinical parameters for the evaluation of dental implants. Periodontol 2000.

[13] Sung Y.T., Wu J.S. (2020). The Visual Analogue Scale for Rating, Ranking and Paired-Comparison (VAS-RRP): A new technique for psychological measurement. Behav. Res. Methods.

[14] Oliveira M.R., Gonçalves A., Gabrielli M.A.C., de Andrade C.R., Vieira E.H., Pereira-Filho V.A. (2021). Evaluation of Alveolar Bone Quality: Correlation Between Histomorphometric Analysis and Lekholm and Zarb Classification. J. Craniofac. Surg..

[15] Manor Y., Oubaid S., Mardinger O., Chaushu G., Nissan J. (2009). Characteristics of early versus late implant failure: A retrospective study. J. Oral Maxillofac. Surg. Off. J. Am. Assoc. Oral Maxillofac. Surg..

[16] Han H.J., Kim S., Han D.H. (2014). Multifactorial evaluation of implant failure: A 19-year retrospective study. Int. J. Oral Maxillofac. Implants.

[17] Howe M.S., Keys W., Richards D. (2019). Long-term (10-year) dental implant survival: A systematic review and sensitivity meta-analysis. J. Dent..

[18] Moraschini V., Poubel L.A., Ferreira V.F., Barboza Edos S. (2015). Evaluation of survival and success rates of dental implants reported in longitudinal studies with a follow-up period of at least 10 years: A systematic review. Int. J. Oral Maxillofac. Surg..

[19] Gomes G.H., Misawa M.Y.O., Fernandes C., Pannuti C.M., Saraiva L., Huynh-Ba G., Villar C.C. (2018). A systematic review and meta-analysis of the survival rate of implants placed in previously failed sites. Braz. Oral Res..

[20] Quaranta A., Cicconetti A., Battaglia L., Piemontese M., Pompa G., Vozza I. (2012). Crestal bone remodeling around platform switched, immediately loaded implants placed in sites of previous failures. Eur. J. Inflamm..

[21] Ferrari Cagidiaco E., Carboncini F., Parrini S., Doldo T., Nagni M., Uti N., Ferrari M. (2018). Functional Implant Prosthodontic Score of a One-Year Prospective Study on Three Different Connections for Single-Implant Restorations. J. Osseointegr..

[22] D’Orto B., Polizzi E., Nagni M., Tetè G., Capparè P. (2022). Full Arch Implant-Prosthetic Rehabilitation in Patients with Type I Diabetes Mellitus: Retrospective Clinical Study with 10 Year Follow-Up. Int. J. Environ. Res. Public Health.

[23] Minervini G., Fiorillo L., Russo D., Lanza A., D’Amico C., Cervino G., Meto A., Di Francesco F. (2022). Prosthodontic Treatment in Patients with Temporomandibular Disorders and Orofacial Pain and/or Bruxism: A Review of the Literature. Prosthesis.

[24] Ciancaglini R., Gherlone E.F., Radaelli G. (1999). Association between loss of occlusal support and symptoms of functional disturbances of the masticatory system. J. Oral Rehabil..

[25] Morton D., Gallucci G., Lin W.S., Pjetursson B., Polido W., Roehling S., Sailer I., Aghaloo T., Albera H., Bohner L. (2018). Group 2 ITI Consensus Report: Prosthodontics and implant dentistry. Clin. Oral Implants Res..

[26] Mello C.C., Lemos C.A.A., Verri F.R., Dos Santos D.M., Goiato M.C., Pellizzer E.P. (2017). Immediate implant placement into fresh extraction sockets versus delayed implants into healed sockets: A systematic review and meta-analysis. Int. J. Oral Maxillofac. Surg..

[27] Eazhil R., Swaminathan S.V., Gunaseelan M., Kannan G.V., Alagesan C. (2016). Impact of implant diameter and length on stress distribution in osseointegrated implants: A 3D FEA study. J. Int. Soc. Prev. Community Dent..

[28] Chrcanovic B.R., Kisch J., Albrektsson T., Wennerberg A. (2018). Factors influencing the fracture of dental implants. Clin. Implant Dent. Relat. Res..

[29] He J., Shang Y.W., Deng C.F., Shang D.H., Zhang C., Wang D.N., Zhao B.H. (2014). A clinical retrospective analysis of dental implants replaced in previously failed sites. Shanghai Kou Qiang Yi Xue.

[30] Machtei E.E., Mahler D., Oettinger-Barak O., Zuabi O., Horwitz J. (2008). Dental implants placed in previously failed sites: Survival rate and factors affecting the outcome. Clin. Oral Implants Res..

[31] Yu T., Gao H., Liu T., Huang Y., Wang C. (2020). Effects of immediately static loading on osteointegration and osteogenesis around 3D-printed porous implant: A histological and biomechanical study. Mater. Sci. Eng. C Mater. Biol. Appl..

[32] Zhang Z.Y., Meng T., Chen Q., Liu W.S., Chen Y.H. (2018). Retrospective analysis of early dental implant failure. Beijing Da Xue Xue Bao Yi Xue Ban.

[33] Liu B.L., Sun H.M., Fang S.H. (2013). Investigation on the psychology of patients with repeated implantation failure and the intervention measures. Jilin Yi Xue.

